# Complex effects on Ca_V_2.1 channel gating caused by a *CACNA1A* variant associated with a severe neurodevelopmental disorder

**DOI:** 10.1038/s41598-022-12789-y

**Published:** 2022-06-02

**Authors:** Benjamin J. Grosso, Audra A. Kramer, Sidharth Tyagi, Daniel F. Bennett, Cynthia J. Tifft, Precilla D’Souza, Michael F. Wangler, Ellen F. Macnamara, Ulises Meza, Roger A. Bannister

**Affiliations:** 1 Departments of Pathology/Biochemistry and Molecular Biology, University of Maryland School of Medicine, 108 North Greene Street, Room 208A, Baltimore, MD 21201 USA; 2 Medical Scientist Training Program, Department of Neurology, Yale School of Medicine, New Haven, CT 06520 USA; 3 National Institutes of Health Undiagnosed Diseases Program, Common Fund, National Institutes of Health, Bethesda, MD 20892 USA; 4 National Human Genome Research Institute, National Institutes of Health, Bethesda, MD 20892 USA; 5 Department of Molecular and Human Genetics, Baylor College of Medicine, Houston, TX 77030 USA; 6 Department of Physiology and Biophysics, School of Medicine, Autonomous University of San Luis Potosí, Carranza #2405, SLP 78210 San Luis Potosí, Mexico; 7 Present Address: Center for Scientific Review, Division of Neuroscience, Development and Aging, National Institutes of Health, 6701 Rockledge Drive, Bethesda, MD 20892 USA

**Keywords:** Diseases of the nervous system, Ion channels in the nervous system

## Abstract

P/Q-type Ca^2+^ currents mediated by Ca_V_2.1 channels are essential for active neurotransmitter release at neuromuscular junctions and many central synapses. Mutations in *CACNA1A*, the gene encoding the principal Ca_V_2.1 α_1A_ subunit, cause a broad spectrum of neurological disorders. Typically, gain-of-function (GOF) mutations are associated with migraine and epilepsy while loss-of-function (LOF) mutations are causative for episodic and congenital ataxias. However, a cluster of severe Ca_V_2.1 channelopathies have overlapping presentations which suggests that channel dysfunction in these disorders cannot always be defined bimodally as GOF or LOF. In particular, the R1667P mutation causes focal seizures, generalized hypotonia, dysarthria, congenital ataxia and, in one case, cerebral edema leading ultimately to death. Here, we demonstrate that the R1667P mutation causes both channel GOF (hyperpolarizing voltage-dependence of activation, slowed deactivation) and LOF (slowed activation kinetics) when expressed heterologously in tsA-201 cells. We also observed a substantial reduction in Ca^2+^ current density in this heterologous system. These changes in channel gating and availability/expression manifested in diminished Ca^2+^ flux during action potential-like stimuli. However, the integrated Ca^2+^ fluxes were no different when normalized to tail current amplitude measured upon repolarization from the reversal potential. In summary, our findings indicate a complex functional effect of R1667P and support the idea that pathological missense mutations in Ca_V_2.1 may not represent exclusively GOF or LOF.

## Introduction

Ca^2+^ flux into nerve terminals via P/Q type (Ca_V_2.1) currents is the initiating event for neurotransmitter release at neuromuscular junctions and many central synapses^1–5^. Like other high voltage-activated Ca_V_ family channels, Ca_V_2.1 is composed by a primary α_1_ and auxiliary α_2_δ and β subunits. The Ca_V_2.1 α_1A_ subunit is a single polypeptide consisting of four highly-conserved membrane-bound repeats (I–IV) connected by three less-conserved intracellular loops (I–II, II–III and III–IV) and bookended by intracellular amino- and carboxyl-termini^6,7^. Each repeat is composed of six transmembrane α-helices (S1–S6). The S5 and S6 helices form structures which support ion selectivity and permeation while the S1–S4 α-helices house the elements of the channel which are responsible for voltage-sensitivity (i.e., the voltage-sensing module)^6,8–10^. The S4 α-helices are considered the primary voltage-sensing components within each module^8,11–13^. When the plasma membrane becomes depolarized, the S4 helix rotates towards the extracellular milieu within an aqueous conduit formed by the S1–S3 helices^14,15^. The outward movement of the S4 helices is dependent on five or six evenly-distributed basic residues lining one face of each S4 helix^8,16–18^. These arginine and lysine residues facilitate translocation by forming transient electrostatic interactions with acidic residues in the S2 and S3 α-helices^17–19^. Translocation of one or more of the S4 helices is coupled to further conformational rearrangements which open the channel pore, though the contributions of each repeat to pore opening are heterogenous and differ amongst Ca_V_ channels^20–24^.

Missense point mutations in *CACNA1A* have long been known to cause Familial Hemiplegic Migraine type 1 (FHM1)^25–28^ and Episodic Ataxia type 2 (EA2)^29,30^. Typically, FHM1-causing mutations are characterized as gain-of-function (GOF)^31,32^ and EA2-causing mutations almost always display loss-of-function (LOF)^33–37^. While FHM1 and EA2 are relatively narrowly-defined classes of disorders, a subset of patients harboring missense mutations in Ca_V_2.1 present with migraine and/or ataxia as well as one or more of the following symptoms: congenital ataxia, congenital encephalopathies, epilepsy, developmental delay, stroke, injury-induced coma and/or respiratory failure^38–51^. The overlapping presentations associated with these more severe disorders are in line with the idea that the impact of pathological missense mutations, or in some cases deletions, in Ca_V_2.1 cannot always be defined exclusively as GOF or LOF^52–56^.

A pathological arginine to proline substitution in the S4 helix of Ca_V_2.1 Repeat IV (R1667P) was first reported by Gauquelin and colleagues^57^. The patient suffered from focal seizures, generalized hypotonia, dysarthria, congenital ataxia and cerebral edema, which lead ultimately to death. In this study, we report a second de novo case of a child with the R1667P variant who presented with global developmental delays, microcephaly, pontocerebellar hypoplasia, and progressive cerebellar ataxia. In addition, we have utilized an open-source model^58^ and whole-cell patch-clamp electrophysiology to characterize the mechanistic consequences of the R1667P mutation on Ca_V_2.1 function. We have found that the R1667P mutation causes both channel GOF (hyperpolarizing voltage-dependence of activation, slowed deactivation) and LOF (slowed activation kinetics) as well as a substantial reduction in Ca^2+^ current density.

## Results

### The Ca_V_2.1 R1667P variant

The Ca_V_2.1 R1667P variant was identified earlier in a female child with focal seizures, generalized hypotonia, dysarthria, congenital ataxia and fatal cerebral edema^57^. We now report a second female child carrying the R1667P variant who presented with global developmental delay, microcephaly, pontocerebellar hypoplasia, thinning of the corpus collosum, small cerebellum, brainstem and pons. In both cases, the arginine to proline substitution was a consequence of a de novo guanine to cytosine mutation at bp 5000 of the *CACNA1A* coding sequence.

### Clinical presentation

The proband is a 6-year, 10-month-old female product of a 37-week in-vitro fertilization, twin gestation. The pregnancy was complicated by an intrauterine bleed at 5 weeks and premature contractions at 35 weeks. She was born via scheduled Caesarean section and was discharged with no post-natal concerns. Birth weight was 6 lbs, 8 oz and she was 18.8 inches long. Her fraternal twin sister is developmentally normal. At four months old she was diagnosed with severe hypotonia, a brain MRI found pontocerebellar hypoplasia, a thin appearing corpus callosum and enlargement of the extra-axial subarachnoid spaces; her electroencephalogram (EEG) was normal. Currently, she is unable to crawl or walk but can be propped to sit. A sleep study at 15 months showed severe obstructive sleep apnea and moderate central apnoea.

Upon examination at 3 years and 10 months, she was found to have dysmorphic features including a large face with long palpebral fissures, prominent epicanthal folds, wide nasal bridge, a high and narrow palate and micrognathia. Growth failure was a significant concern with weight 10.3 kg (< 3%), height 82 cm (3–10%), head circumference 46.5 cm (6% -1SD) and Body Mass Index for age (10–25%). She had a well-balanced, varied diet and her nutrition labs were all normal, though she has chronic constipation.

Awake eye exam revealed normal eye structure, post-surgery strabismus, gaze induced nystagmus, and possible mild cortical visual impairment. Dilated sedated eye exam revealed bilateral hyperopia, optical coherence tomography showed normal macular architecture, however, the peripheral retina had mottled pigment changes. Further neurological examination demonstrated generalized low muscle tone, generalized intentional tremors, with truncal instability and truncal ataxia. Extended EEG showed asymmetric posterior dominant rhythm, vertex waves and spindles with right less than left suggestive of right sided focal dysfunction but no epileptiform discharges. The sensory and motor nerve testing did not reveal evidence of neuropathy or a primary muscle disorder. She had a normal hearing for speech and pure tones and normal auditory brainstem response bilaterally.

A developmental evaluation showed varying degrees of delay in all developmental areas. An MRI at this time showed marked atrophy of the cerebellum and pons with progression since her first MRI at 6 months of age. There was also delayed myelination in the cerebellum with gliotic white matter changes. A magnetic resonance spectroscopy demonstrated deficits of *N*-acetyl aspartate in the pons and superior cerebellar vermis.

### Modelling the R1667P mutation

**Figure 1:**
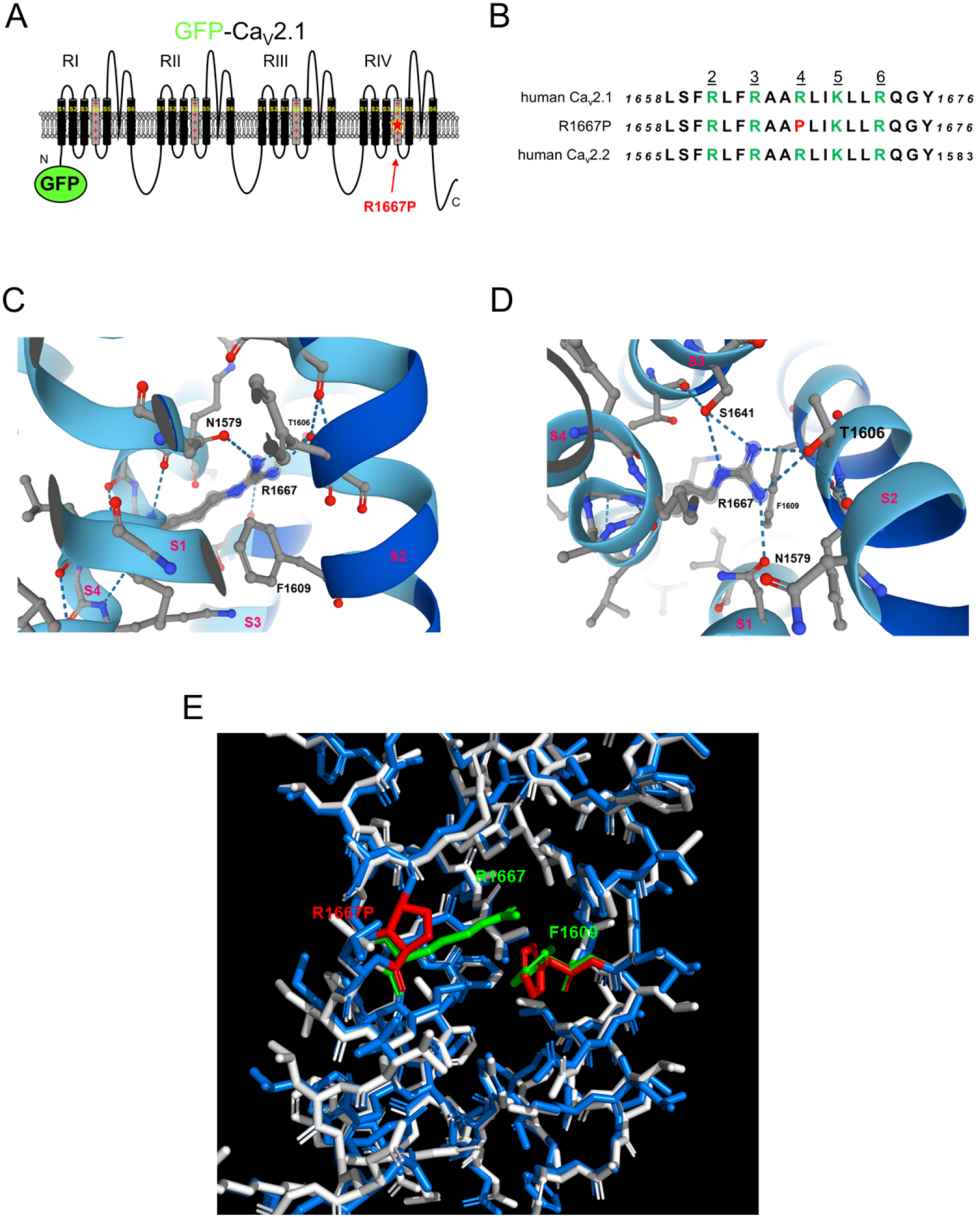
The R1667P mutation occurs at the R4 position of the Repeat IV S4 voltage-sensing α-helix. (**a**) Schematic representation of Ca_V_2.1 with Green Fluorescent Protein (GFP) fused to the amino-terminus (GFP-Ca_V_2.1). The R to P substitution at residue 1667 is indicated by the red star. (**b**) Sequence comparison of the Repeat IV S4 helices of all known human Ca_V_2.1 variants (*cf*. accession no. NP_001120693.1), human Ca_V_2.1 with the R to P substitution at the R4 position and human Ca_V_2.2 (accession no. NM_001243812). Basic residues in positions R2-R6, as defined by Ca_V_2.2 Cryo-EM structure^10^ are shown in green and the R4 R to P substitution is shown in red. (**c**, **d**) AlphaFold2 modeling of the voltage-sensing module of Repeat IV of human Ca_V_2.1^58^. The S1–S4 helices are viewed from the lateral aspect (**c**) and from an extracellular (**d**) vantage points. Potential hydrogen bonds between R1667 and N1579 in S1, T1606 in S2, and S1641 in S3 are indicated by the blue dashed lines. F1609 (i.e., the gating charge transfer center) is also labelled. Panels (**c**) and (**d**) were published with permission https://creativecommons.org/licenses/by/4.0/ (**e**) Missense 3D model showing the impact of the arginine to proline substitution at position 1667. The mutant stick structure (blue) is overlaid on the wild-type stick structure (white) with R1667 shown in green and the R1667P substitution shown in red. In both cases, F1609 is shown; this residue is colored green and red in the in the wild-type and mutant structures, respectively.

The R1667P mutation resides in the “R4” position in the voltage-sensing S4 α-helix of Ca_V_2.1 Repeat IV^6,10^ (Fig. 1a). This R4 residue is highly-conserved amongst vertebrate Ca_V_ channels including the closely-related N-type Ca_V_2.2 channel^13^ (Fig. 1b). Initially, Alphafold2 modelling was used to establish the orientation of R1667 within the Ca_V_2.1 Repeat IV voltage-sensing module (Fig. 1c)^58^. In general, the AlphaFold2 model is consistent with the recent cryo-EM structure of Ca_V_2.2 in which the Repeat IV voltage-sensor is shown in the “up” position^10^. The comparison with the Ca_V_2.2 structure demonstrated that R1667 is positioned just extracellular to a highly-conserved phenylalanine (F1609) in the S2 helix in the open state. F1609 putatively constitutes the isoelectric border between the intra- and extra-cellular compartments of the Repeat IV voltage-sensing module by dividing the aqueous conduit formed by the S1-S3 helices around the S4 helix^59^ (Fig. 1c). AlphaFold2 analysis also inferred that R1667 likely makes hydrogen bonds with N1579 in S1, T1606 in S2, and S1641 in S3 (Fig. 1d). To predict how introduction of a proline at position 1667 might affect these interactions, a homology model based on the Ca_V_2.2 cryo-EM structure was generated using Phyre2. The arginine to proline substitution at position 1667 was introduced using the software Missense 3D (Fig. 1e). This second modelling strategy indicated that the proline: (1) causes a 65º bend in the S4 α-helix, and (2) lacks the positively-charged side chain which extends into the aqueous conduit. The former observation implies a steric “kink” in the voltage-sensor, while the latter suggests the loss of electrostatic contacts with N1579, T1606 and S1641.

### R1667P reduces peak current density but shifts activation to more hyperpolarizing potentials

**Figure 2:**
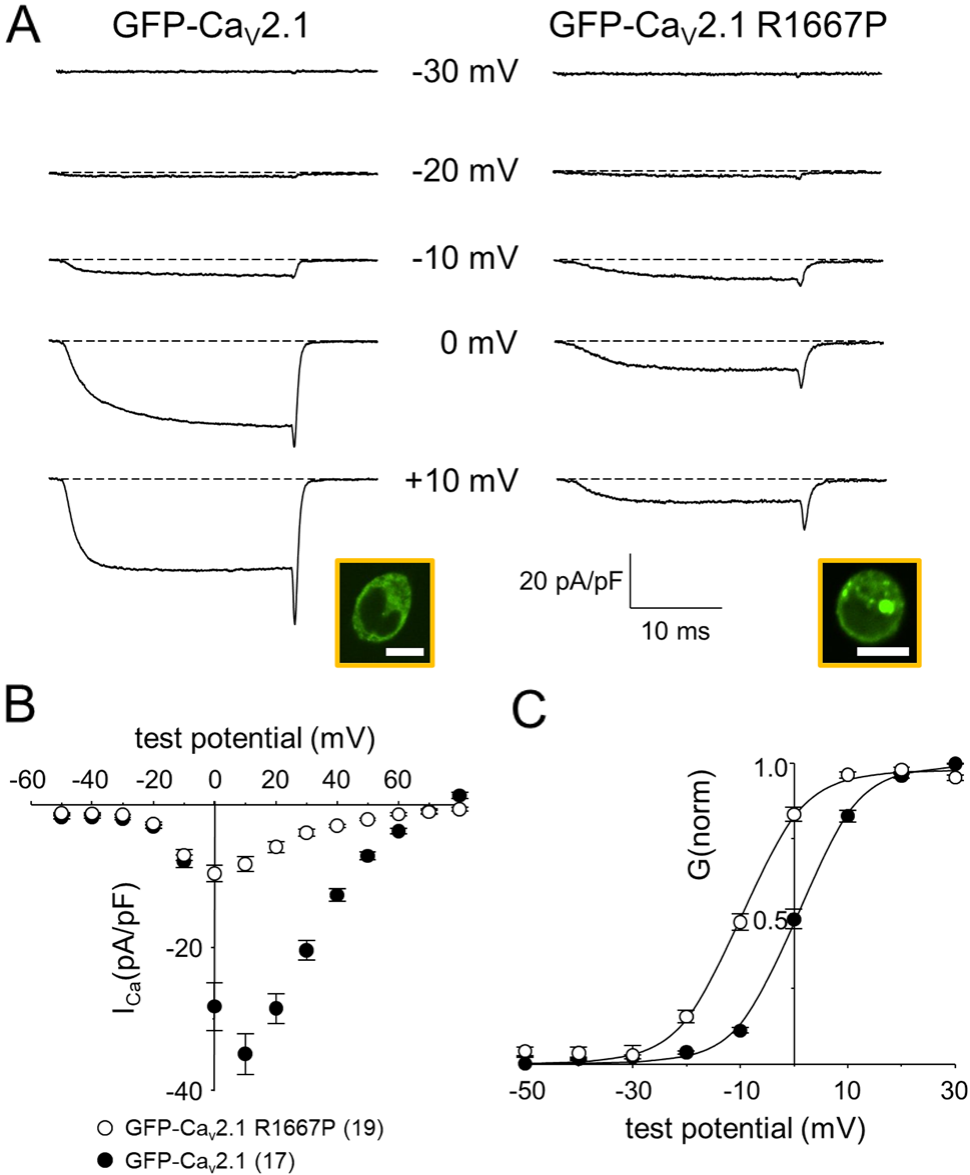
The R1667P mutation causes a profound reduction in Ca^2+^ current density and a hyperpolarizing shift in Ca_V_2.1 activation. (**a**) Ca^2+^ current families recorded from tsA-201 cells expressing GFP-Ca_V_2.1 (*left*) or GFP-Ca_V_2.1 R1667P (*right*) with auxiliary β_4_ and α^2^ δ-1 subunits. Currents were elicited by a 25 ms step depolarizations from − 80 mV to indicated test potentials; the repolarization voltage was − 40 mV. Confocal images confirming successful heterologous expression of GFP-Ca_V_2.1 and GFP-Ca_V_2.1 R1667P are shown in the *insets*. Scale bars = 10 µm. (**b**) Comparison of GFP-Ca_V_2.1 (filled circle; n = 17) and GFP-Ca_V_2.1 R1667P (open circle; n = 19) average peak I–V relationships. Currents were evoked at 0.1 Hz by test potentials ranging from − 50 mV through + 80 mV in 10 mV increments. Amplitudes were normalized by capacitance (pA/pF). (**c**) Normalized G–V curves were fit by Eq. (1) with the following respective parameters for GFP-Ca_V_2.1 and GFP-Ca_V_2.1 R1667P: V_G_ = 0.9 ± 0.7 and − 10.0 ± 0.9 mV; k = 5.4 ± 0.2 and 6.6 ± 0.8 mV, respectively. Throughout, data are presented as mean ± SEM; the total number of cells in a data set is indicated in parentheses.

To investigate the impact of the R1667P mutation on the biophysical properties of Ca_V_2.1, whole-cell Ca^2+^ currents were recorded from tsA-201 cells co-expressing either GFP-fused wild-type Ca_V_2.1 or GFP- fused Ca_V_2.1 carrying the R1667P mutation (GFP-Ca_V_2.1 and GFP-Ca_V_2.1 R1667P, respectively) with auxiliary β_4_ and α_2_ δ-1 subunits. In this set of experiments, GFP-Ca_V_2.1 and GFP-Ca_V_2.1 R1667P both supported inward currents when near physiological 2 mM Ca^2+^ was used as the charge carrier (Fig. 2a). A comparison of the Ca^2+^ current–voltage (I–V) relationships for GFP-Ca_V_2.1 and GFP-Ca_V_2.1 R1667P revealed a considerable decrease in peak current density for the mutant channel (I_peak_ = − 34.9 ± 2.8 pA/pF at + 10 mV, n = 17 vs. − 9.6 ± 1.2 pA/pF at 0 mV, n = 19, respectively; P = 5.5 × 10^–10^) (Fig. 2b). Tail current amplitudes evoked by repolarization from + 70 to − 40 mV were substantially reduced in cells expressing GFP-Ca_V_2.1 R1667P compared to cells expressing GFP-Ca_V_2.1 (I_tail_ = − 15.5 ± 1.8 pA/pF *vs*. − 67.8 ± 5.6 pA/pF, respectively; P = 2.1 × 10^–10^) consistent with the idea that fewer mutant channels are present in the plasma membrane in our heterologous system. Notably, a ~ 10 mV hyperpolarizing shift in activation was evident in both the I-V and the normalized conductance-voltage (G-V) relationships (V_G_ = 0.9 ± 0.7 mV *vs*. − 10.0 ± 0.9 mV for GFP-Ca_v_2.1 and GFP-Ca_V_2.1 R1667P, respectively; P = 1.4 × 10^–10^) (Fig. 2c).

### The R1667P mutation slows activation and deactivation kinetics

**Figure 3:**
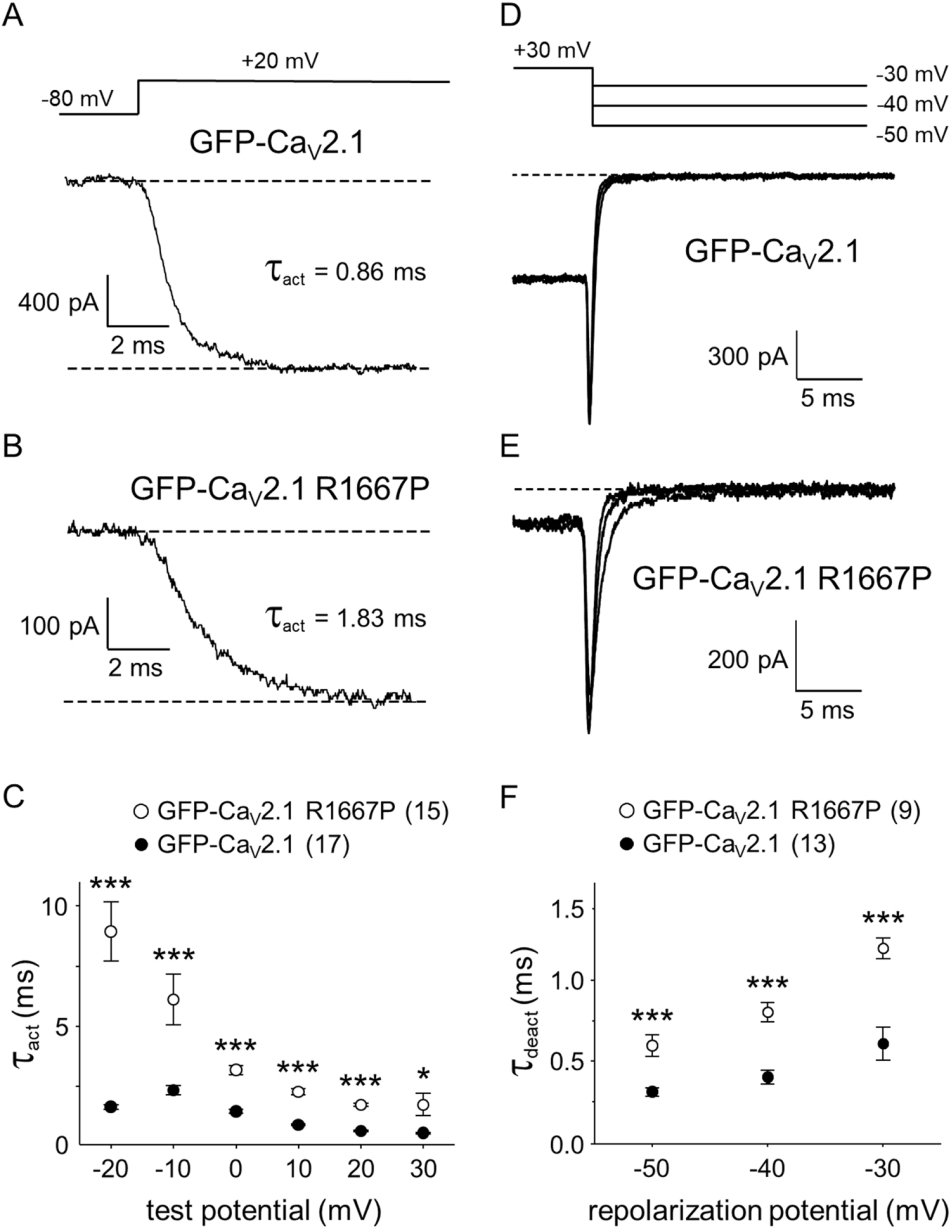
The R1667P mutation slows activation and deactivation. Ca^2+^ currents were recorded from tsA-201 cells expressing either GFP-Ca_V_2.1 (**a**) or GFP-Ca_V_2.1 R1667P (**b**). Currents were elicited by 25 ms step depolarizations from − 80 mV to test potentials ranging from − 20 mV through + 30 mV (**a**-*top*). Activation was fit by Eq. (2); the time constants of activation (τ_act_) for representative cells are indicated. (**c**) Comparison of τ_act_ for GFP-Ca_V_2.1 (filled circle; n = 17) or GFP-Ca_V_2.1 R1667P (open circle; n = 15) measured at the indicated test potentials. Representative tail currents were recorded from tsA-201 cells expressing either GFP-Ca_V_2.1 (**d**) or GFP-Ca_V_2.1 R1667P (**e**) upon repolarization from + 30 mV to the indicated potentials (**d**-top). Deactivation was fit by Eq. (2). (**f**) Comparison of τ_deact_ for GFP-Ca_V_2.1 (filled circle; n = 13) and GFP-Ca_V_2.1 R1667P (open circle; n = 9) measured at the indicated repolarization potentials. Significant differences by two-tailed, unpaired t-test are indicated (*Denotes P < 0.05; ***Denotes P < 0.001).

A closer examination of GFP-Ca_V_2.1 and GFP-Ca_V_2.1 R1667P current families indicated that GFP-Ca_V_2.1 R1667P activated much more slowly than GFP-Ca_V_2.1 (τ_act_ at + 20 mV = 0.7 ± 0.0 ms, n = 17 *vs*. 1.8 ± 0.1 ms, n = 15, respectively, P = 4.0 × 10^–15^) (Fig. 3a–c). Similarly, the R1667P mutation also impaired deactivation (Fig. 3d,e); analysis of tail currents evoked by repolarization steps from + 30 mV to a range of increasingly more positive potentials beginning at -50 mV yielded slower rates of channel closure (τ_deact_) for GFP-Ca_V_2.1 R1667P relative to GFP-Ca_V_2.1 at all repolarization potentials (all P < 0.0002) (Fig. 3f). Defects in voltage- and/or Ca^2+^-dependent inactivation may also exist, but rigorous investigation of inactivation from the open state was precluded by the slow activation kinetics of the currents in cells expressing GFP-Ca_V_2.1 R1667P.

### R1667P has little effect on closed-state inactivation

**Figure 4:**
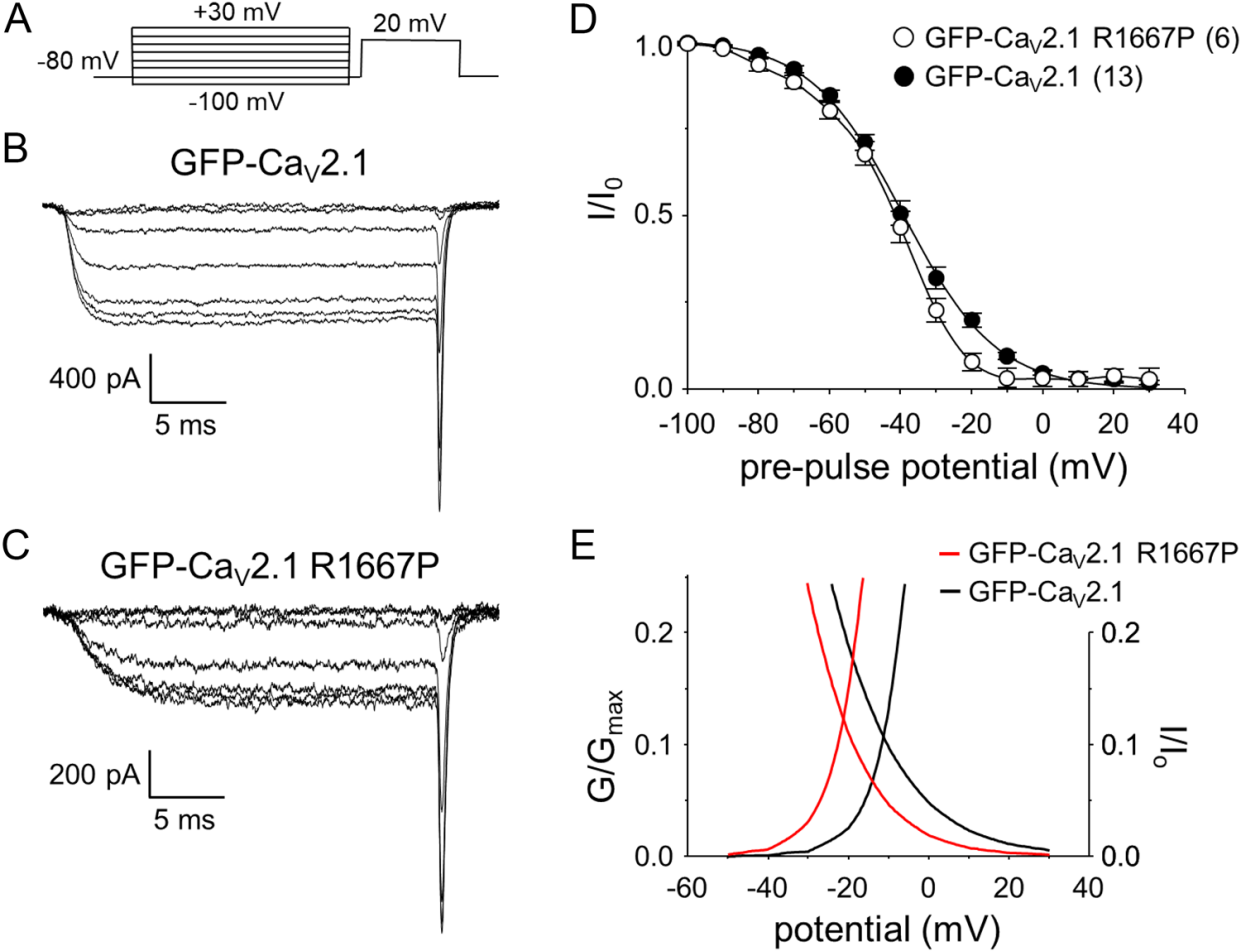
The R1667P mutation has little effect on closed-state inactivation. (**a**) A 5 s conditioning step from the steady holding potential (− 80 mV) to increasing potentials ranging from − 100 to + 30 mV (in 10 mV increments) was applied before repolarizing the membrane to − 80 mV for 5 ms. Test currents were then evoked by a 25 ms step depolarization to + 20 mV. The protocol is not drawn to scale. Representative Ca^2+^ currents recorded from tsA-201 cells expressing GFP-Ca_V_2.1 (**b**) or GFP-Ca_V_2.1 R1667P (**c**) after pre-pulses to − 100, − 80, − 60, − 40, − 20, 0 and + 20 mV. Normalized steady-state inactivation curves for GFP-Ca_V_2.1 (filled circle; n = 13) and GFP-Ca_V_2.1 R1667P (open circle; n = 6) are shown in (**d**); amplitudes were normalized by the maximal Ca^2+^ current in each cell. The normalized inactivation relationships were fit with Eq. (3) with the following fit parameters for GFP-Ca_V_2.1 and GFP-Ca_V_2.1 R1667P: V_1/2inact_ = − 39.3 ± 15 and − 42.1 ± 1.8 mV; k = − 12.6 ± 0.6 and − 10.7 ± 0.7 mV, respectively. (**e**) Overlay of the smooth conductance and closed-state inactivation curves (from Figs. 2c and 4d, respectively) for cells expressing GFP-Ca_V_2.1 (black lines) and GFP-Ca_V_2.1 R1667P (red lines).

Five second conditioning steps were employed to assess channel closed-state inactivation (illustrated in Fig. 4a). The conditioning step was followed immediately by a 5 ms return to – 80 mV before a 25 ms depolarizing test step to + 20 mV. This protocol yielded no clear differences between GFP-Ca_V_2.1 (Fig. 4b) and GFP-Ca_V_2.1 R1667P (Fig. 4c). Specifically, the normalized inactivation relationships for GFP-Ca_V_2.1 and GFP-Ca_V_2.1 R1667P had similar half-inactivation potentials (V_1/2inact_ = − 39.3 ± 1.5 mV; n = 13 *vs*. − 42.1 ± 1.8 mV, n = 6, respectively; P = 0.30) (Fig. 4d) and slope values (k = − 12.6 ± 0.6 mV *vs*. − 10.7 ± 0.7 mV, respectively; P = 0.087). There was virtually no change in the magnitude of the window current (2.31 *vs*. 2.45 arbitrary units for GFP-Ca_V_2.1 and GFP-Ca_V_2.1 R1667, respectively). However, the window current range was shifted to more hyperpolarizing potentials (Fig. 4e) as a consequence of the ~ 10 mV hyperpolarizing shift in activation for GFP-Ca_V_2.1 R1667P (Fig. 2c).

### R1667P reduces Ca^2+^ flux in response to action potential-like stimuli

**Figure 5:**
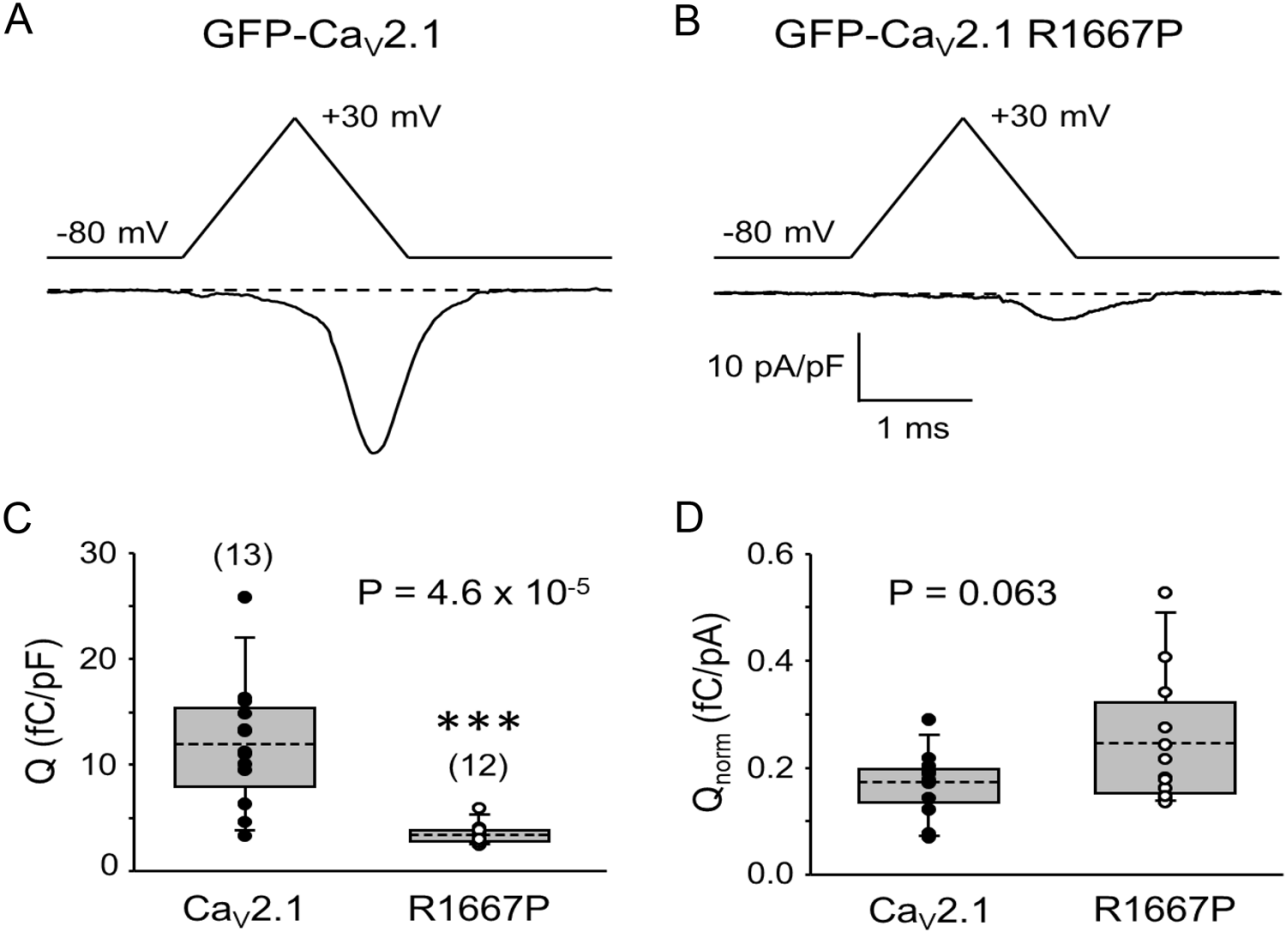
The R1667P mutation reduces total Ca^2+^ flux in response to a single action potential-like stimulus. Ca^2+^ currents attributable to GFP-Ca_V_2.1 (**a**) and GFP-Ca_V_2.1 R1667P (**b**) were evoked by an action potential-like waveform consisting of a 1 ms ramp from − 80 mV to + 30 mV followed immediately by a 1 ms ramp back to − 80 mV. Traces shown are the average of 10 recordings. (**c**) Comparison of total charge flux normalized to cell membrane capacitance (fC/pF) for cells expressing GFP-Ca_V_2.1 (filled circle; n = 13) or GFP-Ca_V_2.1 R1667P (open circle; n = 12). (**d**) Absolute charge flux normalized to tail current amplitude at the reversal potential (i.e., maximal conductance) (fC/pA). Means are indicated by the dashed lines of the boxes. Boxes represent the 25th/75th percentiles. Bars represent the 5th/95th percentiles. A significant difference is indicated (***) in (**c**).

The R1667P substitution shifts activation and, consequently, the window current to more hyperpolarizing potentials (Figs. 2, 4). The mutation also retards deactivation (Fig. 3). These effects imply that the R1667P manifests in channel GOF under physiological circumstances. By contrast, the reduced current density and slower activation kinetics suggest that R1667P may result in overall channel LOF (Figs. 2, 3). To investigate these effects of the mutation in a somewhat more physiological context, we evoked Ca^2+^ flux via an action potential-like voltage-clamp protocol similar to that used by Bahamonde et al.^54^. Robust Ca^2+^ influx via GFP-Ca_V_2.1 was observed using this protocol (Q = 12.0 ± 1.6 fC/pF; n = 13) (Fig. 5a) but the total integrated charge flux for GFP-Ca_V_2.1 R1667P was found to be much less (3.4 ± 0.3 fC/pF, n = 12; P = 4.6 × 10^–5^) (Fig. 5b).

The disparity in current density between cells expressing GFP-Ca_V_2.1 and GFP-Ca_V_2.1 R1667P (Fig. 2) raised the possibility that the observed reduction in Ca^2+^ flux (Fig. 5c) was a consequence of fewer functional channels resident in the plasma membrane. To assess this possibility, the total integrated charge flux in each cell (i.e., Q) was normalized to maximal whole-cell conductance (i.e., the amplitude of the tail current upon repolarization from the reversal potential to − 40 mV) to reasonably estimate flux via individual channels. Per this metric, the Ca^2+^ fluxes supported by individual GFP-Ca_V_2.1 and GFP-Ca_V_2.1 R1667P channels were not significantly different (Q/I_tail_ = 0.17 ± 0.02 fC/pA, n = 13 *vs*. 0.25 ± 0.04 fC/pA, n = 12, respectively; P = 0.063) (Fig. 5d). However, flux could vary between the wild-type and mutant channels during repetitive use.

## Discussion

In this study, we identified a second patient carrying the pathological *CACNA1A* R1667P variant and characterized the biophysical impact of the mutation on Ca_V_2.1 channel function. In tsA-201 cells expressing GFP-fused Ca_V_2.1 R1667P, we observed a substantial reduction in current density (Fig. 2) and multiple alterations in gating including a ~ 10 mV hyperpolarizing shift in activation (Fig. 2), slowed activation kinetics (Fig. 3a–c) and slowed deactivation kinetics (Fig. 3d–f). Intuitively, one would expect alterations in gating considering that R1667 is a basic residue in the “R4” position of one of the channel’s four S4 voltage-sensing α-helices^13,60^. Our models showed that, in the putative open-state, R1667 is located just extracellular to the Repeat IV S2 phenylalanine (F1609) which represents the isoelectric point, or gating charge transfer center, of the channel^59^. The introduction of a proline at this position was predicted to partially disrupt the helical nature of the S4 helix (Fig. 1c,d) and to preclude key hydrogen bonds of R1667 with N1579, T1606, and S1641 in S1, S2 and S3, respectively (Fig. 1e). The ablation of these molecular contacts provides a plausible explanation for the hyperpolarizing shift in activation whereby stabilization of the Repeat IV S4 helix with the R4 position extracellular to the gating charge transfer center (i.e., F1609) favours a “primed” conformation. By the same token, we posit that the inability of R1667P to efficiently form hydrogen bonds with N1579, T1606 and/or S1641 slightly impedes voltage-sensor return and subsequent channel closure upon repolarization (Fig. 3d–f).

On the surface, the increased activation of the current at more hyperpolarizing membrane potentials (Fig. 2) and slower activation kinetics (Fig. 3) of the R1667P mutant seem counterintuitive. A potential explanation for the incongruence is that introduction of the proline has two independent effects on gating. First, the “priming” of the Repeat IV voltage-sensor shifts activation to more hyperpolarizing potentials (please see above). Second, the proline-induced 65° twist in the S4 α-helix may impose a steric impediment to later, voltage-independent transitions critical for opening of the pore, as has been observed previously for a proline substitution at the R6 position in Repeat IV^61^. Thus, the R1667P mutation seems to precipitate both GOF and LOF in the same channel.

The hyperpolarized activation and slower rate of deactivation would together serve to increase Ca^2+^ entry into synaptic terminals^54,62^, whereas the slower activation kinetics would almost certainly promote a reduction in neurotransmission^63^. However, the opposing effects of the mutation on channel gating appeared to effectively cancel (Fig. 5d), leaving the reduction in current density as the primary determinant in reducing total Ca^2+^ flux during single action potential-like stimuli (Fig. 5c). If the GOF and LOF effects largely cancel in situ and the net effect of R1667P on unitary Ca^2+^ flux is effectively minimal, the explanation that mutation simply impairs axon terminal Ca^2+^ influx through haploinsufficiency becomes a tempting conclusion. That said, a more sophisticated experimental model (e.g., iPSCs, knock-in animals) is required to rigorously test the hypothesis that R1667 precludes expression/trafficking of the channel in neurons. The importance of such approaches cannot be overstated as channel behaviour can differ between tsA-201 cells and neurons^53^. Without such information, caution should be exercised when drawing conclusions regarding the systemic impact of the observed changes in voltage-dependence and expression on systemic pathology.

The complex presentation of the two patients carrying the R1667P mutation may be related to a perturbation of the delicate balance of GOF and LOF effects on the channel^57^. Two other patients, a mother and daughter, have been identified with an arginine to tryptophan substitution at the same position (R1667/8W)^39^. The mother presented with progressive cerebellar ataxia, ocular deficiencies and cerebellar atrophy. The daughter shared these characteristics but also displayed migraine and symptoms more consistent with EA2. Some of these attributes were common with the two non-related R1667P patients. However, neither the seizure symptoms nor cerebral edema associated with the first case of the R1667P mutation were present within the R1667/8W family suggesting that overall effects of the proline introduction are more disruptive of normal channel function than introduction of a tryptophan.

The generalization that GOF mutations in Ca_V_2.1 are associated with migraine while LOF mutations are causative for episodic ataxia is usually correct^31–37^, though exceptions have been documented^30,64,65^. Moreover, the phenotypes of Ca_V_2.1 channelopathies can be spectral and differ in degree of severity^38–51^. In some of these rapidly presenting cases, there is an almost stroke-like presentation^66–68^. For many of the subset of disorders which include these overlapping characteristics, both GOF and LOF effects on Ca_V_2.1 function have been reported for the same mutation. Early on, Pietrobon and colleagues demonstrated that the primarily FHM1-linked mutations increased channel P_o_ but also produced lower current amplitude owing to a lower number of functional channels in the membrane^52,53^. In particular, the widely-studied S218L variant displayed paradoxical increased voltage-sensitivity and decreased current density^69^. Likewise, a pathogenic Ca_V_2.1 variant missing a phenylalanine at position 1502 had a substantial LOF (reduced peak current) which was overridden by strong GOF (~ 20 mV hyperpolarizing shift in activation and slowed deactivation) during action potential-mimicking stimuli^54^. More recently, Gandini et al.^56^ demonstrated that the Y1384C mutation causes both channel GOF (increased window current) and LOF (reduced current density) in tsA-201 cells. Thus, our current study adds to a growing body of work that supports the idea that increased voltage-sensitivity and decreased current density are an electrophysiological signature of a subset of severe Ca_V_2.1 channelopathies which feature both ataxia and migraine.

## Methods

### Compliance with ethics of experimentation

The current study was approved by Institutional Review Board of The National Institutes of Health, National Human Genome Research Institute (15-HG-0130). Informed consent for publication was obtained and is on record. The study was performed in accordance with the principles of the Declaration of Helsinki. No animals were used in this study.

### Health and safety

All laboratory procedures were approved by University of Maryland Baltimore Environmental Health and Safety (00005440).

### Modeling

The AlphaFold2 model of human Ca_V_2.1 can be accessed at the following web address: https://alphafold.ebi.ac.uk/entry/O00555^58^. Homology models of full length Ca_V_2.1 α_1A_ subunits were generated using Phyre2 employing Ca_V_2.2 α_1B_ as a template^70^. The sequence of Ca_V_2.1 α_1A_ was downloaded from the universal protein resource (Uniprot; entry: O00555). The optimal template for homology modelling was identified using the BLASTp program^71^. The 3D structure of Ca_V_2.2 α_1B_ (Uniprot ID: Q00975) was downloaded from PDB (PDB ID:7miy) and served as the template structure. The secondary structure of Ca_V_2.1 α_1A_ was predicted using Phyre2. Missense mutation analysis was performed using Missense 3D^72,73^. Structures were visualized in PyMol.

### Molecular biology

The GFP-Ca_V_2.1 R1667P mutant construct was derived from the plasmid GFP-Ca_V_2.1, known alternatively as EGFP-FLAG-α_1A_ BI-1 (V1)^74^. To generate GFP-Ca_V_2.1 R1667P, a guanine to cytosine substitution at bp 5000 of the sequence encoding Ca_V_2.1 (Δ10A (− V + G), 16^+^17^+^, Δ17A (− VEA), − 31* (− NP), 37a (EFa), 43^+^44^+^, Δ47) was introduced into the plasmid by Genscript, Inc. using proprietary methods. The integrity of the novel construct was guaranteed by Genscript, Inc. and confirmed by restriction digests and sequencing upon receipt.

### Cell culture and transfection

tsA-201 cells (American Type Culture Collection) were cultured and transfected using Lipofectamine 2000 (Invitrogen) as described previously^61^. The transfection mixture contained expression plasmids encoding GFP-Ca_V_2.1 or GFP-Ca_V_2.1 R1667P with rat β_4_ and rabbit α_2_ δ-1 auxiliary subunits (1 µg of each cDNA per 35 mm well). The day following transfection, cells were trypsinized, re-plated onto 35 mm Primaria-treated plastic culture dishes (BD Falcon) and transferred to a 30 °C humified incubator. Successfully transfected (i.e., GFP-positive) cells were used in experiments ~ 24 h later.

### Fluorescence imaging

Images of live tsA-201 cells were acquired using a Nikon W1 Spinning Disk microscope. Briefly, GFP was excited with a 488 nm line. The emitted GFP fluorescence was directed to the detector via a 500–550 nm band-pass filter. Similar detector gains were used for both sets of images.

### Whole-cell patch clamp electrophysiology

All electrophysiological experiments were performed at room temperature (21–25 °C). Borosilicate pipettes (2.5–4.0 MΩ) were filled with an internal solution containing (mM): 140 Cs-Aspartate, 10 Cs_2_-EGTA, 5 MgCl_2_ and 10 HEPES, pH 7.4 with CsOH. The external solution contained (mM): 145 tetraethylammonium-Cl, 2 CaCl_2_, 10 HEPES, 10 glucose and pH 7.4 with tetraethylammonium-OH. Currents were recorded with an Axon 200B patch amplifier and digitized via a Digidata 1550 analog-to-digital converter (both Molecular Devices). Electronic compensation was used to reduce the effective series resistance. Linear components of leak and capacitive currents were corrected with − P/4 online subtraction protocols. Filtering was at 5–10 kHz and digitization was at 50–100 kHz. Cell capacitance (C_m_) was determined by integration of a transient from − 80 to − 70 mV using Clampex 10.6 (Molecular Devices) without compensation (23.0 ± 0.8 pF, n = 36) and was used to normalize current amplitudes (pA/pF). The time constant for decay of the whole-cell capacity transient (τ_m_) was reduced as much as possible using the analog compensation circuit of the amplifier. The values of τ_m_ and access resistance (R_a_) were 108.8 ± 8.9 µs and 4.9 ± 0.2 MΩ respectively.

Conductance–voltage (G–V) relationships were obtained from the I_tail_–V data where individual tail current amplitudes evoked from a given test potential were normalized by the maximal tail current amplitude produced by repolarization to – 40 mV. Normalized I_tail_ values were subsequently fit with the equation:

1 $${\text{G}}/{\text{G}}_{{{\text{max}}}} = { 1}/\left( {{1 } + {\text{ exp}}\left( { - \left( {{\text{V}}_{{\text{G}}} - {\text{ V}}} \right)/{\text{k}}} \right)} \right)$$

where G is the tail current amplitude evoked by repolarization from a given test potential back to − 40 mV, G_max_ is the maximal tail current amplitude obtained, V_G_ is the half-maximal activation potential, and k is the slope factor. Activation and deactivation recordings were fit with a single exponential function:

2 $${\text{I}} = {\text{I}}_{0} ({\text{exp}}( - {\text{t}}/\tau ))$$

Normalized closed-state inactivation curves were fit by the equation:

3 $${\text{I}}/{\text{I}}_{0 } = { 1}/\left( {{1 } + {\text{ exp}}\left( { - \left( {{\text{V}}_{{{1}/{\text{2inac}}}} - {\text{ V}}} \right)/{\text{k}}} \right)} \right)$$

where I is the step current amplitude evoked by a 25 ms test depolarization to + 20 mV which was evoked following a 5 s conditioning step to potentials ranging from − 100 mV to + 30 mV and 5 ms return to − 80 mV, I_0_ is the maximal step current amplitude obtained during the protocol, V_1/2inact_ is the half-inactivation potential, and k is the slope factor. Window currents were quantified via integration of the area under the conductance and inactivation curves.

Two-phase ramp waveforms intended to mimic neuronal action potentials were generated using a protocol similar to those used by Bahamonde and colleagues^54^. Cells were depolarized by a 1 ms ramp from − 80 to + 30 mV followed immediately by a 1 ms repolarization ramp to − 80 mV. The total charge flux was calculated as the integral of the area under the current evoked by the ramp protocol. Charge flux in each cell was then divided by the amplitude of the tail current following a repolarization from a 25 ms step to + 70 mV (i.e., near the reversal potential) back to − 40 mV to normalize Ca^2+^ flux to channel expression.

### Analysis

SigmaPlot (version 12.0, Systat Software, Inc.) and Origin software (version 8.0, Microcal Software, Inc.) were used for data analysis and for construction of figures. All data are presented as mean ± SEM. Statistical comparisons were made by unpaired, two-tailed t-test, with P < 0.05 considered significant.

## Data Availability

The datasets generated and/or analysed during the current study are publicly available in the ClinVar, https://www.ncbi.nlm.nih.gov/clinvar/variation/638582/?new_evidence=false (accession number VCV000638582.1); ModelArchive https://modelarchive.org/doi/10.5452/ma-oynl4 (accession number ma-oynl4) and AlphaFold2, https://alphafold.ebi.ac.uk/entry/O00555 (accession number O00555) databases. The AlphaFold2 structure was based on https://www.uniprot.org/uniprot/O00555 (UniProt accession number O00555). All other datasets obtained during the present study are available from the corresponding author upon reasonable request.
